# Relationship Between Nurses’ Second Victim Experience and Professional Identity: The Mediating Role of Psychological Resilience

**DOI:** 10.3390/healthcare14203439

**Published:** 2026-10-14

**Authors:** Dalin Kuang, Jiangying Han, Xiaoyun Zhao, Yinhua Zhang, Ruiqi Wang, Xiaomin Zhang, Xingqiao Tao, Peng Zhu

**Affiliations:** 1 School of Public Health, Anhui Medical University, No. 81 Mei Shan Road, Hefei 230032, China; yfy224301@fy.ahmu.edu.cn; 2 Department of Nursing, The First Affiliated Hospital of Anhui Medical University, Hefei 230022, China; yfy284569@fy.ahmu.edu.cn (J.H.); yfy2651128@fy.ahmu.edu.cn (X.Z.); yfy228185@fy.ahmu.edu.cn (Y.Z.); yfy6641093@fy.ahmu.edu.cn (R.W.); yfy2651433@fy.ahmu.edu.cn (X.Z.); yfy2651532@fy.ahmu.edu.cn (X.T.)

**Keywords:** nurse, second victim experience, psychological resilience, professional identity, exploratory cross-sectional mediation analysis

## Abstract

**Highlights:**

**What are the main findings?**

- In this cross-sectional study, nurses who had experienced nursing adverse events reported a mean second victim experience total score of 55.03 ± 12.42, suggesting a noticeable level of second victim-related burden; psychological resilience and professional identity were both at moderate levels.
- A significant association was observed between second victim experience and professional identity. Mediation analysis revealed that psychological resilience played a statistically significant role in the indirect relationship between second victim experience and professional identity, with the nature of the mediation effect differing across the total score and specific dimensions of second victim experience.

**What are the implications of the main findings?**

- Psychological resilience may be a potential intervention target for nurses with second victim experiences, and the dimension-specific findings suggest that support-related aspects of these experiences may also deserve attention rather than focusing only on distress.
- Nursing managers may consider combining resilience-oriented components with organizational support strategies, such as psychological counseling, peer support, reflective debriefing, mentorship, and supportive team responses after nursing adverse events. Because these findings reflect cross-sectional statistical associations rather than causal evidence, their practical value should be further examined in longitudinal and interventional studies.

**Abstract:**

Background/Objectives: The second victim phenomenon is increasingly recognized in patient safety, yet the pathways connecting second victim experience to nurses’ professional outcomes remain unclear. This study aimed to investigate the relationships among second victim experience, psychological resilience, and professional identity, and to examine the mediating role of psychological resilience. Methods: This study used convenience sampling to recruit nurses who had experienced nursing adverse events at a tertiary Grade-A hospital. Participants were assessed using the Second Victim Experience and Support Tool, the Connor–Davidson Resilience Scale, and a Professional Identity Scale. Data were analyzed using SPSS 25.0, with Pearson/Spearman correlation and mediation analyses performed. Results: A total of 368 nurses who experienced nursing adverse events were included in the study. Scores for second victim experience, professional identity, and psychological resilience were 55.03 ± 12.42, 113.82 ± 21.42, and 28.04 ± 7.33, respectively. The mean second victim experience distress and support dimension (reverse-scored) scores were 34.33 ± 10.21 and 20.70 ± 6.84, respectively; their correlation was small and non-significant (r = 0.023, *p* = 0.722). In the total-score model, the indirect effect of second victim experience on professional identity through psychological resilience was significant (indirect effect = −0.308, 95% CI [−0.400, −0.223]), whereas the direct effect was not (direct effect = −0.080, *p* = 0.158). In the support-dimension model, both the indirect effect (indirect effect = −0.211, 95% CI [−0.290, −0.143]) and the direct effect (direct effect = −0.341, *p* < 0.001) were significant. In the distress-dimension model, the indirect effect was negative and significant (indirect effect = −0.227, 95% CI [−0.318, −0.138]), whereas the direct effect was positive and significant (direct effect = 0.128, *p* = 0.012), resulting in a non-significant total effect (total effect = −0.099, 95% CI [−0.222, 0.023]). Conclusions: The Second Victim Experience and Support Tool composite score reflects multiple facets of the second victim experience, encompassing both distress-related and support-related components. Perceived support deficit was consistently associated with lower professional identity, partly through psychological resilience, whereas distress showed a more complex indirect association through resilience. These findings suggest that psychological resilience may be a potential intervention target and that both distress- and support-related aspects of second victim experience warrant attention. However, because this was a cross-sectional mediation analysis, the findings should be interpreted as statistical associations rather than causal relationships.

## 1. Introduction

Nursing practice is characterized by high risk, high pressure, and high complexity. Consequently, despite continuous improvements in healthcare quality management and patient safety in recent years, nursing adverse events (NAEs) remain difficult to completely eliminate. NAEs refer to unplanned and typically undesirable incidents that occur during the delivery of nursing care. Such events not only cause direct harm to patients and their families but may also exert significant psychological and professional impacts on the healthcare professionals involved [1]. In medical adverse events, patients and their families are commonly regarded as the first victims, whereas healthcare professionals who are drawn into the event and suffer psychological and emotional trauma are referred to as second victims (SVs). The concept of the “SV” was first introduced by Wu in 2000 to describe healthcare professionals who experience fear, guilt, self-blame, and psychological trauma due to concerns about the consequences following adverse medical events [2]. Subsequently, Scott et al. (2009) further defined SV as healthcare providers who are traumatized by involvement in unanticipated adverse events, medical errors, or near misses [3]. In clinical nursing practice, nurses serve as the direct implementers of patient treatment and care, having the most frequent contact with patients and bearing substantial professional responsibilities; therefore, they are prone to becoming SVs following NAE [4]. In the present study, SV experience refers specifically to the psychological, physical, and professional distress experienced by nurses following their involvement in NAEs during clinical practice, including—but not limited to—medication errors, patient falls, pressure injuries, unplanned extubation, and other patient safety incidents. Prior research has demonstrated that nurses frequently exhibit sustained emotional dysregulation, self-doubt, intrusive recollections of the incident, and apprehension regarding peer evaluation following NAE. In severe cases, manifestations may include anxiety, depression, insomnia, and burnout, a constellation of symptoms collectively termed “SV syndrome” [5,6,7].

As SVs, the impact on clinical nurses extends beyond the psychological domain and may further permeate into professional development. Professional identity represents the comprehensive evaluation of nursing professionals regarding the value of the nursing profession, professional roles, responsibilities, and sense of belonging, serving as a critical foundation for maintaining professional commitment, work engagement, and professional stability [8]. If nurses remain in a prolonged state of negative emotions and self-denial following NAEs, their positive appraisal of the nursing profession may be undermined, leading to diminished professional identity and even precipitating turnover intention and burnout [9]. Existing research indicates that approximately 18.5% to 31.8% of nurses experiencing the SV phenomenon harbor turnover intentions, suggesting that this issue not only pertains to individual psychological adaptation but is also closely related to nursing workforce stability and quality of care assurance [10]. Both the National Nursing Development Plan (2021–2025) and the Healthy China 2030 Planning Outline explicitly emphasize the importance of attending to nurses’ mental health, optimizing the structure of the nursing talent pool, and enhancing overall workforce stability [11,12]. However, despite the documented impact of the SV experiences on turnover intention and professional stability, there remains a paucity of research examining its relationship with professional identity—a critical determinant of professional commitment, work engagement, and long-term workforce retention. Therefore, exploring the relationship between SV experiences and professional identity is of substantial significance for understanding nurses’ psychological recovery and occupational well-being.

The pathways connecting SV experience to professional identity remain poorly understood. Lazarus and Folkman’s transactional theory of stress and coping offers a valuable framework for elucidating this relationship [13]. According to this theory, an individual’s response to a stressor (such as an SV experience) is determined by two interrelated psychological processes: cognitive appraisal and coping [13]. Psychological resilience, defined as an individual’s dynamic capacity to positively reappraise adverse circumstances and deploy effective coping strategies when confronted with stress, adversity, or trauma, represents the adaptive manifestation of these processes [14]. Previous studies have demonstrated that psychological resilience is closely associated with SV-related distress and has also been associated with nurses’ professional outcomes [15]. Higher psychological resilience may be associated with more adaptive appraisal of NAEs, less self-blame and negative rumination, stronger stress adjustment, and greater professional functioning and confidence [15]. The existing evidence base provides a strong rationale for hypothesizing psychological resilience as a mediator. As such, psychological resilience may serve as a critical mediator through which SV experiences influence professional identity. Compared with other positive psychological constructs such as self-efficacy, optimism, or social support, psychological resilience is uniquely suited as a mediator in this context because it is a dynamic capacity that directly reflects the stress appraisal and coping processes theorized by the transactional model, rather than a static personality trait or an external resource. Additionally, psychological resilience plays a vital role in coping with emergencies, buffering negative emotions, and facilitating psychological recovery, and it is a modifiable factor that can be cultivated through targeted interventions. If psychological resilience is confirmed to mediate the SV experience–professional identity relationship, this would provide a clearer understanding of the pathways through which SV experiences may relate to professional identity, which could inform future hypothesis-driven research on resilience-related support for nurses following NAEs.

**Figure 1:**
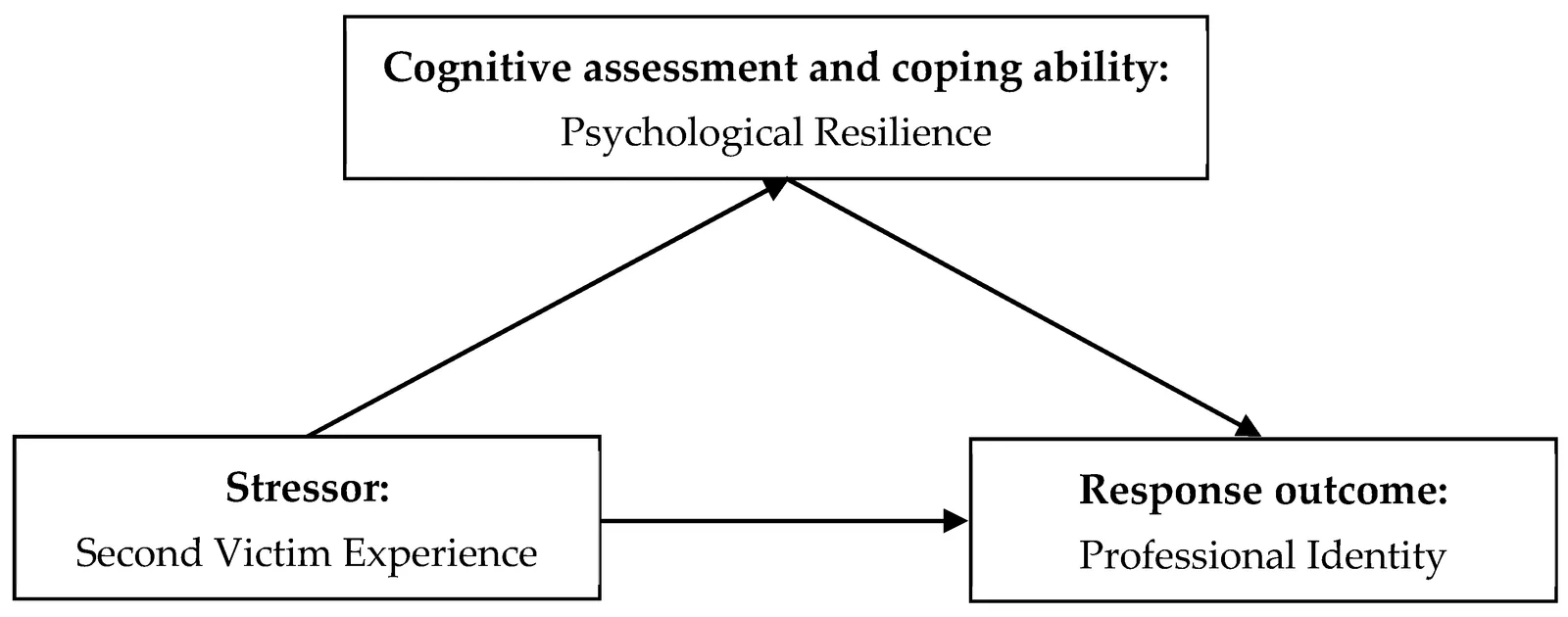
Hypothesis model: relationship between Second Victim Experience and Professional Identity.

Existing research on SVs has predominantly focused on prevalence rates and associated risk factors. While a growing body of literature has begun to examine the psychological sequelae and career outcomes among nurses, these studies have largely centered on negative endpoints such as turnover intention and job burnout. Scant attention has been paid to professional identity—a positive occupational psychological construct encompassing both cognitive evaluation and emotional belonging. Moreover, prior investigations have remained largely at the level of direct-effect testing, failing to illuminate the potential indirect pathways linking SV experiences to professional outcomes. In particular, the mediating role of psychological resilience in this relationship has yet to be systematically explored. Against this background, the present study was designed to investigate the status of SV experience, psychological resilience, and professional identity among nurses experienced NAEs, and to examine the interrelationships among these three variables, the hypothesized model is illustrated in Figure 1. This study used the Second Victim Experience and Support Tool (SVEST) to assess nurses’ SV experiences. The total score reflects overall burden and allows comparison with previous studies. However, because it combines two distinct components, we also analyzed the distress and support dimensions separately and in mutually adjusted models to examine whether they showed different associations with professional identity. This approach provides both an overall estimate and a more nuanced understanding of the distinct roles of the two SVEST dimensions. This study contributes to the literature in three ways: (1) it provides the first empirical test of psychological resilience as a mediator in the SV experience–professional identity relationship; (2) it shifts the focus from turnover intention—a relatively well-studied outcome in the SV literature—to professional identity, which represents a more fundamental and stable cognitive–affective evaluation of professional worth; and (3) it contributes to the literature by offering preliminary insights that, if supported by future longitudinal or interventional research, may eventually inform resilience-building interventions designed to sustain professional identity among nurses experiencing NAEs.

## 2. Materials and Methods

### 2.1. Subjects

This cross-sectional study was conducted at a tertiary hospital in China from January 2025 to January 2026. Registered nurses with SV experience were recruited through convenience sampling. In this study, SV experience was operationalized as the psychological distress experienced by a nurse following direct involvement in a NAE during clinical care. Such distress included fear, guilt, self-blame, or intrusive recollections attributable to the event. Eligible NAEs encompassed medication errors, patient falls, pressure injuries, unplanned extubation, and other patient safety incidents with actual or potential harm. Nurses who merely witnessed an NAE without direct care involvement were not considered to have SV experience. Inclusion criteria included the following: (a) registered nurses who had been directly involved in at least one NAE and self-identified as experiencing psychological distress related to that event; (b) voluntary participation with written informed consent; (c) employment at the hospital as a regular full-time employee for at least one year. Exclusion criteria included the following: (a) absence from clinical duty for more than one month during the data collection period (e.g., due to maternity leave, sick leave, or secondment); (b) failure to complete the questionnaire within the designated period; (c) nurses who only witnessed an NAE without direct involvement in the care process leading to the event; (d) nurses who reported psychological distress related to routine clinical emergencies (e.g., patient deterioration, resuscitation attempts) in the absence of NAEs. The required sample size was estimated a priori using G*Power (ver. 3.1.9.7; Heinrich-Heine-Universität Düsseldorf, Düsseldorf, Germany). For multiple linear regression with a medium effect size (f^2^ = 0.15), a significance level of α = 0.05, and a statistical power of 0.80, the minimum required sample size was 138 [16]. Because mediation analysis requires adequate power to detect indirect effects, which are typically smaller than direct effects, we applied a conservative safety factor of 1.5, yielding a target sample size of approximately 200 [17]. The final sample of 368 nurses exceeded this threshold, providing sufficient statistical power to detect small-to-medium indirect effects. The study was approved by the Ethics Committee of the First Affiliated Hospital of Anhui Medical University (PJ2024-04-60) and all subjects signed the informed consent. In order to guarantee a comprehensive and accurate research reporting, we used the Strengthening the Reporting of Observational Studies in Epidemiology (STROBE) guideline for cross-sectional studies, and all methods were performed in accordance with the relevant guidelines and regulations.

### 2.2. Investigation Questionnaires and Scales

#### 2.2.1. Demographic Questionnaire

The demographic questionnaire was designed by the researchers based on the literature including gender, age, marital status, educational background, working period, professional title, employment status, both time and type of latest experiencing NAEs and inaccessible/accessible to professional psychological counseling.

#### 2.2.2. Second Victim Experience and Support Tool (SVEST)

The Chinese version of SVEST, translated by Chen and colleagues [18], was used to assess the SV experience in this study. This 24-item questionnaire comprises six dimensions: psychological distress, physical distress, practice distress, colleague support, management support, and no work-related support. It is scored by a 5-point Likert scale with anchors ranging from 1 (completely disagree) to 5 (completely agree). Prior to computing the total score, items within the three support-related dimensions were reverse-coded to ensure consistent scoring direction, such that higher scores uniformly indicate less perceived support. The total score was treated as a continuous variable, calculated by summing all item scores after reverse coding, with a theoretical range of 24 to 120. Higher total scores denote more severe SV symptoms and poorer support experiences. The overall Cronbach’s α of the Chinese version of the SVEST was 0.824. The construct validity of the SVEST was established in prior validation research, and we assessed its internal consistency in the current sample. In the present study, Cronbach’s alpha was 0.869 for the overall SVEST, with subscale alphas ranging from 0.734 to 0.908.

#### 2.2.3. Connor–Davidson Resilience Scale (CD-RISC)

Psychological resilience was assessed using the 10-item C-D RISC, originally developed by Campbell-Sills and Stein as a brief version of the 25-item CD-RISC [19]. The Chinese version was translated and validated by Wang et al. among Chinese earthquake survivors [20]. The scale comprises 10 items rated on a 5-point Likert scale ranging from 0 (not true at all) to 4 (true nearly all the time), yielding a total score from 0 to 40, with higher scores indicating greater psychological resilience. The reported Cronbach’s alpha coefficient was 0.91; Cronbach’s alpha in the present sample was 0.964.

#### 2.2.4. Professional Identity Scale for Nurses (PIS)

The PIS is produced by Liu et al. [21], including professional cognitive evaluation (9 items), professional social skills (6 items), professional social support (6 items), professional frustration coping (6 items) and professional self-reflection (3 items). All 30 items were rated on a 5-point Likert scale, maximal threshold of 150, among which, 30–60 indicates a low level, 61–90 indicates a relatively low level, 91–120 indicates a medium level, and 121–150 indicates a high level. The scores were positively associated with professional identity. The reported Cronbach’s alpha coefficient was 0.938. Cronbach’s alpha in the present sample was 0.981.

### 2.3. Data Collection and Statistical Analysis

**Figure 2:**
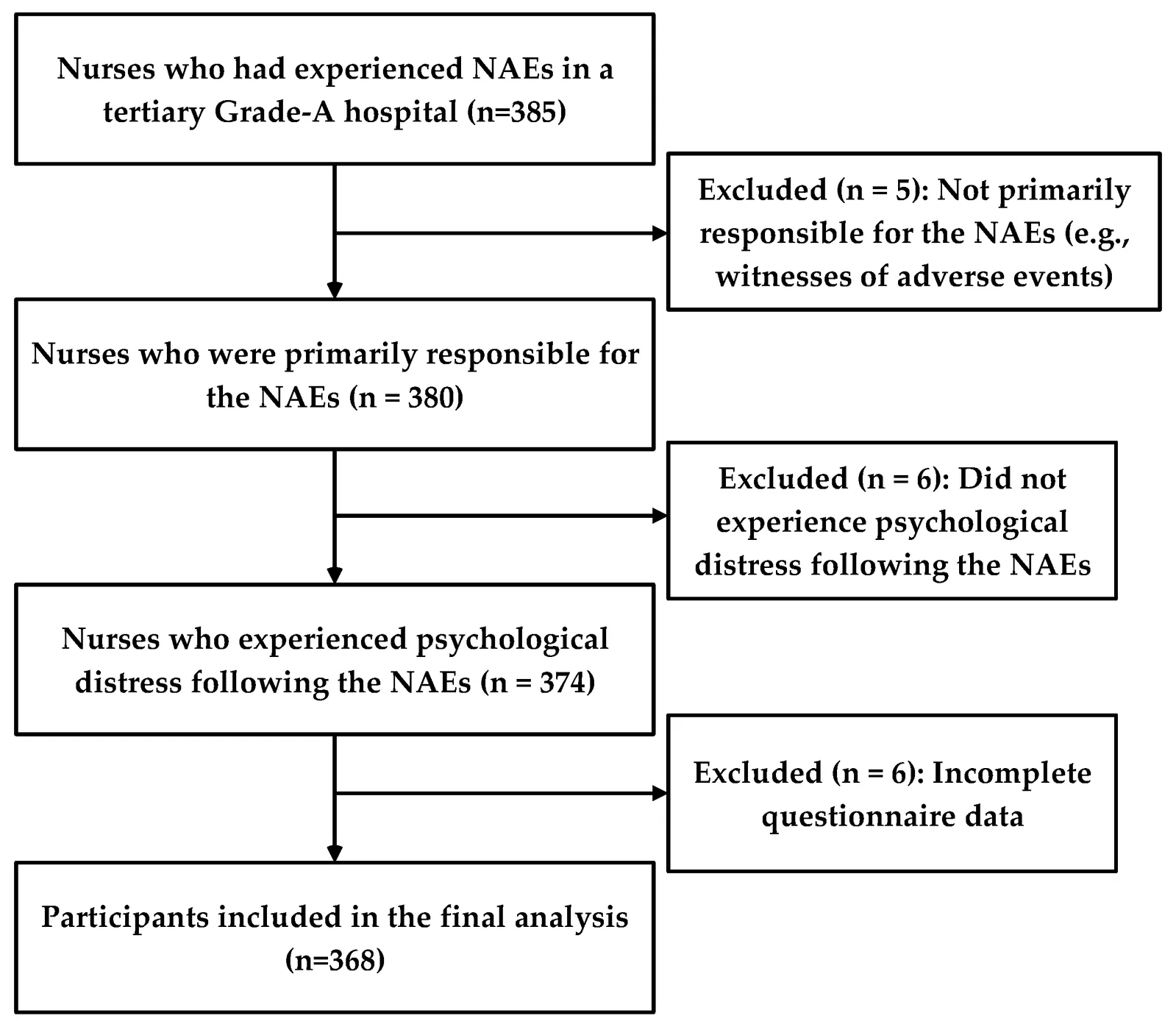
Participant flow diagram. Note: NAEs, nursing adverse events.

Eligible nurses were recruited through departmental meetings, internal hospital notices, and WeChat work groups. Prospective participants first completed a screening item asking whether they had been directly involved in any NAE. Those who responded affirmatively then indicated whether they had experienced psychological distress (e.g., guilt, self-blame, anxiety, or intrusive thoughts) related to that event. Only those meeting both conditions were invited to complete the full survey. The flow of participants through the study is shown in Figure 2. All participants were instructed to respond with reference to their most recent NAE during clinical practice. Data were collected using anonymous, self-administered questionnaires. Prior to distribution, the research objectives and procedures were explained, and standardized instructions were provided to minimize response bias. All responses were collected within a designated timeframe to ensure confidentiality and data integrity.

Data were double-entered using EpiData software (ver. 3.1; The EpiData Association, Odense, Denmark) to ensure accuracy and consistency. Statistical analyses were performed using IBM SPSS Statistics (ver. 25.0; IBM Corp., Armonk, NY, USA). Continuous variables were expressed as mean ± standard deviation (M ± SD), and categorical variables were expressed as frequencies and percentages. Before correlation and regression analyses, the completeness of the analytic variables was examined. Missing data were summarized for each variable, and complete-case analyses were planned; no imputation was performed. The distributions of continuous variables were assessed using histograms, normal probability plots, and skewness and kurtosis statistics. For Pearson correlation analyses, scatterplots were inspected to assess approximate linearity, homoscedasticity, and potential bivariate outliers. Potential univariate outliers were examined using standardized scores. Spearman rank correlations were additionally calculated as a sensitivity analysis to assess whether the correlation pattern was robust to minor departures from normality and the presence of extreme observations. Mediation analyses were conducted using PROCESS macro (ver. 4.2, Model 4; Andrew F. Hayes, Calgary, AB, Canada) [22] with 5000 bootstrap resamples. Bootstrap 95% confidence intervals (CIs) were used to assess indirect effects; an indirect effect was considered statistically significant when its 95% CI did not include zero. Unstandardized coefficients were estimated using the original score scales, and standardized coefficients were calculated after standardizing continuous variables. Linearity and residual behavior were assessed using residual-versus-fitted plots, normal probability plots, and tests of quadratic terms. Homoscedasticity was assessed using the Breusch–Pagan test. Leverage values and Cook’s distance were examined to identify potentially influential observations, and multicollinearity was assessed using variance inflation factors (VIFs). Where assumption diagnostics indicated heteroscedasticity, HC3 heteroscedasticity-consistent standard errors were used in sensitivity analyses.

## 3. Results

### 3.1. General Data of Nurse

A total of 385 questionnaires were distributed to nurse who had experienced NAEs, yielding 368 valid responses (95.58% response rate). Among the 368 nurses who had experienced NAEs as SV, the sample was predominantly female (>90%) and largely comprised young professionals, with ages ranging from 23 to 59 years (average age 32.00 ± 4.55 years) and approximately 90% being under 40 years of age. Most participants held a bachelor’s degree, had intermediate or lower professional titles, and reported no religious affiliation (>90%); additionally, over 60% were married. Regarding employment characteristics, the vast majority had less than 16 years of work experience (89.13%) and were formally employed by both their department and the hospital (98%). With respect to the nature of NAEs, patient falls and catheter dislodgement were the most commonly reported incidents. Moreover, there were 65 subjects (17.66%) had received psychological counseling A statistically significant group difference in professional identity scores was observed between counseling recipients and non-recipients (*p* < 0.01) (Table 1).

### 3.2. Score of SVEST

The average score of SVEST was moderately 55.03 ± 12.42, among which, the score in psychological distress dimension was the highest, 3.71 ± 0.90, moreover, the score of family and friends’ support was the highest in support dimension, 2.14 ± 1.05, which demonstrated that for SV, less support could be obtained from family members and friends. The mean SV experience distress dimension score was 34.33 ± 10.21, and the mean support dimension (reverse-scored) score was 20.70 ± 6.84. The correlation between distress and support (reverse-scored) was small and non-significant (*r* = 0.023, *p* = 0.722), supporting their treatment as related but empirically distinct domains (Table 2).

### 3.3. Score of PIS and CD-RISC

The mean professional identity score among nurse who experienced SV was 113.82 ± 21.42, with the highest subscale score observed in professional self-reflection and the lowest in professional social skills. Additionally, the mean psychological resilience score was 28.04 ± 7.33 (Table 3).

### 3.4. Correlation Analysis Between SV Experience and Psychological Resilience as Well as Professional Identity

All continuous variables were approximately normally distributed (skewness: −0.129 to 0.141), with the exception of the SV experience support dimension (reverse-scored, with higher values indicating greater support deficiency; skewness = 0.814). Scatterplots indicated approximately linear relationships and no marked heteroscedasticity. Four participants had standardized scores >3 on at least one variable; all values were within plausible ranges and were retained. Spearman correlation sensitivity analyses supported the Pearson correlations, which were used for all continuous study scores (Table 4). At the total score level, SV experience was negatively correlated with psychological resilience (*r* = −0.534, *p* < 0.001) and professional identity (*r* = −0.414, *p* < 0.001), while psychological resilience was positively correlated with professional identity (*r* = 0.661, *p* < 0.001). At the dimension level, the SV experience distress dimension showed negative correlation with psychological resilience (*r* = −0.348, *p* < 0.001) and was not significantly correlated with professional identity (*r* = −0.119, *p* = 0.063). The support dimension was negatively correlated with both psychological resilience (*r* = −0.451, *p* < 0.001) and professional identity (*r* = −0.574, *p* < 0.001). (Table 4).

### 3.5. Domain-Specific Mediation Analyses of Psychological Resilience

After standardizing all variables, three separate mediation models were tested using Hayes’s PROCESS macro (Model 4, 5000 bootstrap samples). Psychological counseling (coded 1 = yes, 2 = no; with the higher value indicating the absence of counseling) was included as a covariate based on theoretical considerations and univariate findings, as it reflects differential access to organisational support that may confound the relationships between second-victim experiences and both psychological resilience and professional identity. In the total-score model, SV experience was negatively associated with professional identity (*β* = −0.388, *p* < 0.001); after accounting for psychological resilience, the direct association was attenuated and no longer significant (*β* = −0.080, *p* = 0.158), whereas psychological resilience remained positively associated with professional identity (*β* = 0.599, *p* < 0.001). When dimensions were analyzed separately, divergent patterns emerged. For the SV experience support dimension (reverse-scored), scores were negatively associated with professional identity when psychological resilience was not in the model (*β* = −0.552, *p* < 0.001). After psychological resilience was entered, this direct association was attenuated yet remained significant (*β* = −0.341, *p* < 0.001), while psychological resilience itself was positively associated with professional identity (*β* = 0.491, *p* < 0.001). For the distress dimension, the total association with professional identity was not significant (*β* = −0.099, *p* = 0.112). However, after accounting for psychological resilience, the direct association became significantly positive (*β* = 0.127, *p* = 0.012), whereas resilience remained positively associated with professional identity (*β* = 0.685, *p* < 0.001). Statistically, the indirect effect via resilience was negative and the direct effect was positive; these opposite-sign effects offset each other, resulting in a non-significant total effect. In mutually adjusted models, SV experience distress and support dimensions were both negatively associated with psychological resilience (*β* = −0.327 and −0.426, respectively; both *p* < 0.001). In the final model, support dimension remained negatively associated with professional identity (*β* = −0.328, *p* < 0.001), whereas the direct association between distress dimension and professional identity was not statistically significant after adjustment for support dimension and psychological resilience (*β* = 0.077, *p* = 0.103) (Table 5).

**Table 5:** The multiply regression of variables in mediation effect model.

Regression		Global Fit Index						Significance of Regression Coefficient		
Outcome variables	Prediction variables	B	SE	β	95% CI for B	t	p	R	R^2^	F
SV experience total score as the independent variable										
Professional identity	SV experience	−8.310	1.244	−0.388	−10.760, −5.858	−6.679	<0.001	0.447	0.200	30.368
	Psychological counseling	−10.825	3.691	−0.170	−18.096, −3.556	−2.933	0.004			
Psychological resilience	SV experience	−0.514	0.054	−0.514	−0.621, −0.407	−9.478	<0.001	0.550	0.303	52.791
	Psychological counseling	−0.394	0.161	−0.134	−0.711, −0.077	−2.450	0.015			
Professional identity	SV experience	−1.714	1.210	−0.080	−4.097,0.669	−1.417	0.158	0.671	0.450	66.050
	Psychological resilience	12.834	1.223	0.599	10.425,15.243	10.495	<0.001			
	Psychological counseling	−5.767	3.104	−0.091	−11.881, 0.347	−1.858	0.064			
SV experience support dimension score as the independent variable										
Professional identity	SV experience support dimension	−1.728	0.164	−0.552	−2.051, −1.406	−10.568	<0.001	0.593	0.351	65.795
	Psychological counseling	−9.462	3.320	−0.149	−16.001, −2.923	−2.850	0.005			
Psychological resilience	SV experience support dimension	−0.460	0.061	−0.430	−0.580, −0.339	−7.524	<0.001	0.475	0.226	35.388
	Psychological counseling	−3.239	1.241	−0.149	−5.683, −0.796	−2.611	0.010			
Professional identity	SV experience support dimension	−1.068	0.154	−0.341	−1.371, −0.766	−6.956	<0.001	0.734	0.538	93.940
	Psychological resilience	1.436	0.145	0.491	1.150, 1.722	9.889	<0.001			
	Psychological counseling	−4.811	2.847	−0.076	−10.418, 0.796	−1.690	0.092			
SV experience distress dimension score as the independent variable										
Professional identity	SV experience distress dimension	−0.209	0.131	−0.099	−0.466, 0.049	−1.595	0.112	0.251	0.063	8.160
	Psychological counseling	−14.081	3.962	−0.222	−21.885, −6.276	−3.554	0.001			
Psychological resilience	SV experience distress dimension	−0.238	0.042	−0.331	−0.321, −0.154	−5.592	<0.001	0.393	0.154	22.122
	Psychological counseling	−3.974	1.288	−0.183	−6.510, −1.438	−3.086	0.002			
Professional identity	SV experience distress dimension	0.267	0.106	0.128	0.059, 0.476	2.530	0.012	0.678	0.460	68.691
	Psychological resilience	2.003	0.150	0.685	1.707, 2.299	13.337	<0.001			
	Psychological counseling	−6.121	3.073	−0.096	−12.174, −0.068	−1.992	0.048			
Mutually adjusted models for SV experience distress and support dimensions										
Psychological resilience	SV experience distress dimension	−0.235	0.038	−0.327	−0.309, −0.160	−6.199	<0.001	0.576	0.332	40.036
	SV experience support dimension	−0.456	0.057	−0.426	−0.568, −0.344	−8.021	<0.001			
	Psychological counseling	−2.613	1.159	−0.120	−4.897, −0.330	−2.254	0.025			
Professional identity	SV experience distress dimension	0.162	0.099	0.077	−0.033, 0.356	1.639	0.103	0.737	0.543	71.617
	SV experience support dimension	−1.027	0.155	−0.328	−1.333, −0.722	−6.624	<0.001			
	Psychological resilience	1.530	0.156	0.523	1.223, 1.837	9.826	<0.001			
	Psychological counseling	−4.937	2.838	−0.078	−10.527, 0.653	−1.740	0.083			

Note: NAE, nursing adverse event; SV, Second Victim; B, unstandardized coefficient; SE, model-based standard error; β, standardized regression coefficient; CI, confidence interval. Psychological counseling was coded 1 = yes and 2 = no, with the higher value indicating the absence of counseling. Support dimension: reverse-scored, with higher values indicating greater support deficiency. Intercepts are included but not displayed. HC3 robust-standard-error sensitivity analyses did not change the direction or statistical significance of the key associations. All models adjusted for professional psychological counseling. Mutually adjusted models also controlled for the other SVEST domain. After controlling for psychological counseling, bootstrap analyses (Table 6) revealed three distinct statistical patterns in the association between SV experience and professional identity. In the total-score model, the indirect effect of psychological resilience was negative and statistically significant (indirect effect = −0.308, *95% CI* [−0.400, −0.223]), while the direct effect was not significant (direct effect = −0.080). In the support-dimension model, the indirect effect through psychological resilience was also negative and statistically significant (indirect effect = −0.211, *95% CI* [−0.290, −0.143]), and the direct effect remained statistically significant (direct effect = −0.341). In the distress-dimension model, the indirect effect through psychological resilience was negative and statistically significant (indirect effect = −0.227, *95% CI* [−0.318, −0.138]), whereas the direct effect was positive and statistically significant (direct effect = 0.128). This resulted in a non-significant total effect, suggesting an inconsistent mediation pattern, possibly aligned with statistical suppression. Overall, the statistical patterns indicate that psychological resilience influences the relationship between SV experiences and professional identity, but the nature and strength of this influence vary across dimensions (Table 6, Figure 3). However, due to the cross-sectional design, these findings should be interpreted as associations rather than causal mechanisms.

**Table 6:** The analysis of mediation effect from psychological resilience between SV experience and professional identity.

Independent Variable	Mediation	Effect	Standard Error	*95% CI*	
				Low	Upper
SV experience total score	Direct	−0.080	0.057	−0.191	0.031
	Indirect	−0.308	0.045	−0.400	−0.223
	Total effect	−0.388	0.058	−0.502	−0.274
support dimension	Direct	−0.341	0.049	−0.438	−0.245
	Indirect	−0.211	0.037	−0.290	−0.143
	Total effect	−0.552	0.052	−0.655	−0.449
distress dimension	Direct	0.128	0.050	0.028	0.227
	Indirect	−0.227	0.046	−0.318	−0.138
	Total effect	−0.099	0.062	−0.222	0.023

Note: SV, Second Victim; CI, confidence interval.

**Figure 3:**
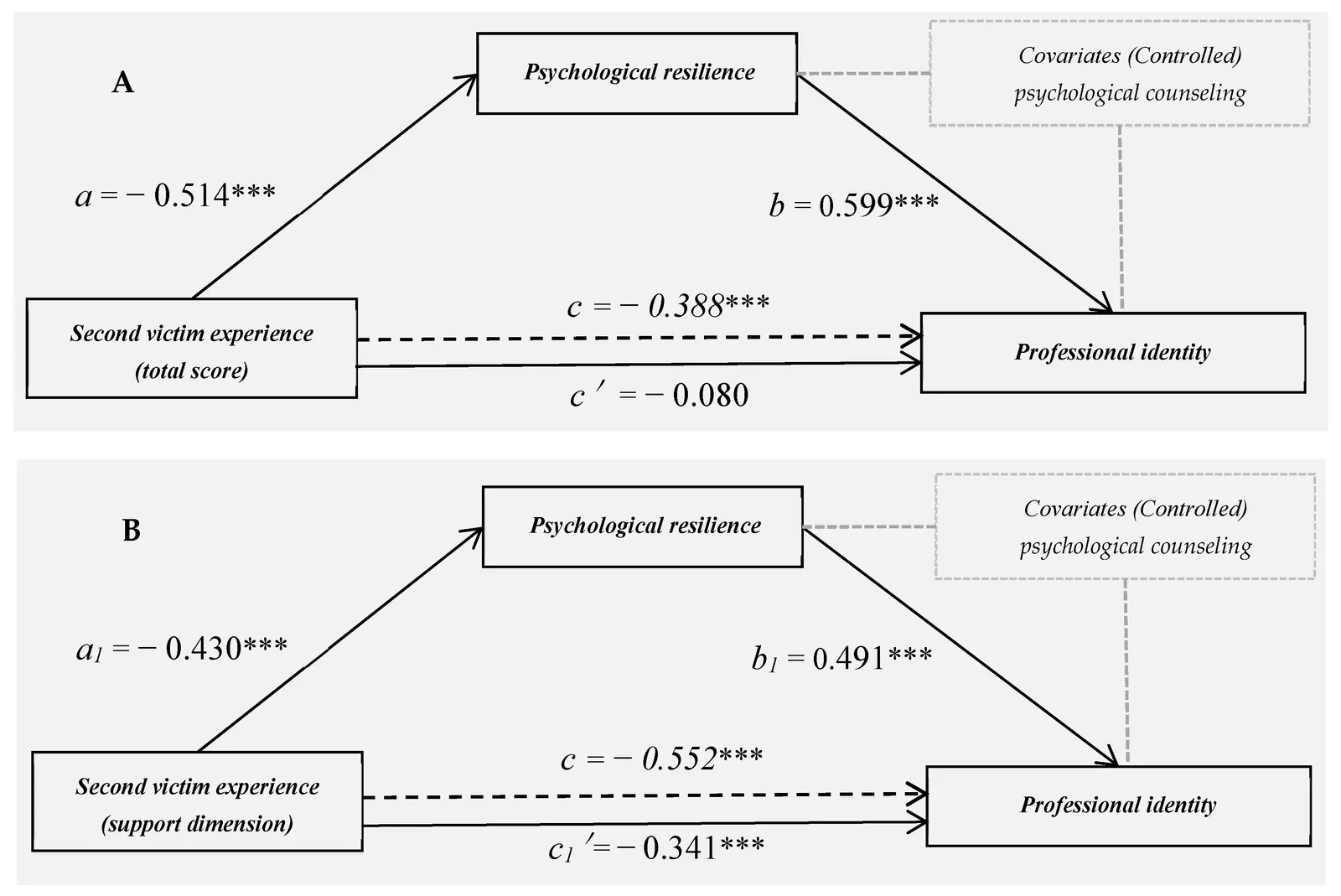

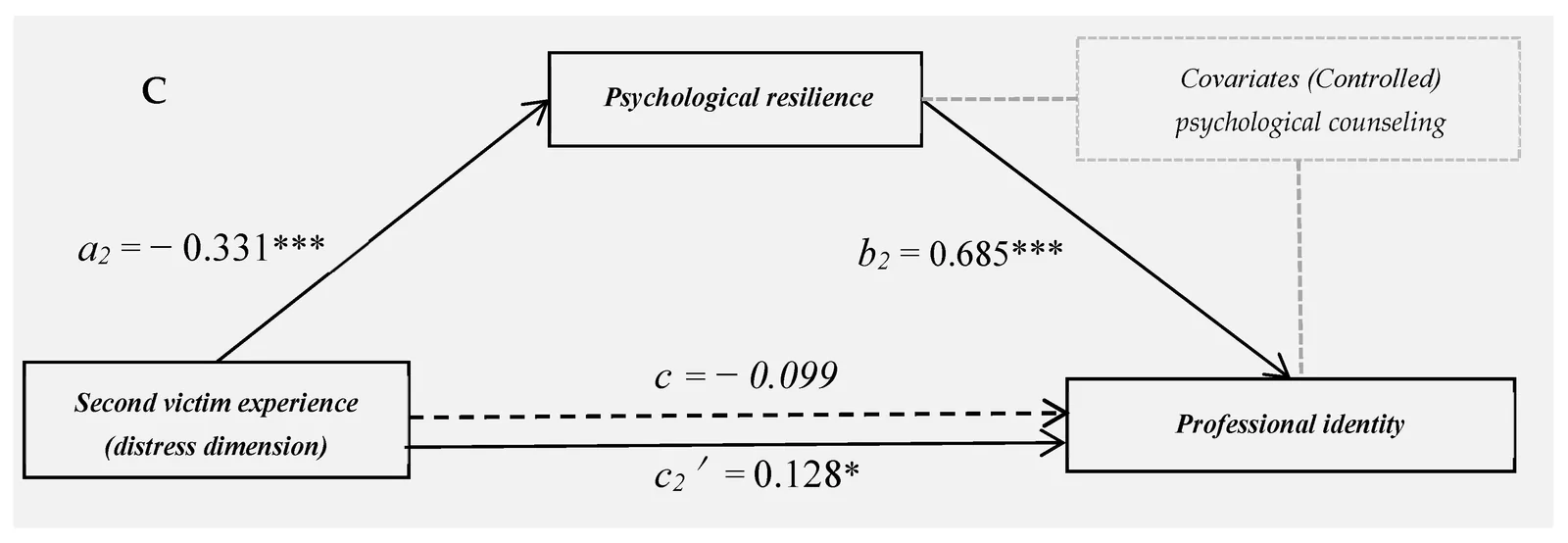
The mediation effect model of psychological resilience between second victim experience and professional identity. Note: Mediation models were tested using Hayes’s PROCESS macro (Model 4) with 5000 boot-strap samples and 95% confidence intervals. (**A**) Total SV experience score; (**B**) Support dimension (re-verse-scored, with higher values indicating greater support deficiency); (**C**) Distress dimension. Path a represents the association between SV experience and psychological resilience; path b represents the association between resilience and professional identity; path c′ represents the direct association between SV experience and professional identity after controlling for resilience; path c represents the total effect. Co-variate (controlled in all paths): psychological counseling. * *p* < 0.05, *** *p* < 0.001. These models are based on cross-sectional data; causal interpretations are precluded.

### 3.6. Regression Diagnostics and Sensitivity Analyses

Regression diagnostics for the mutually adjusted professional identity model indicated no major departure from linearity based on residual-versus-fitted plots, normal probability plots, and the quadratic-term check (*F* = 2.370). Residuals showed mild positive skewness (0.468) and excess kurtosis (0.915). The Breusch–Pagan test indicated mild heteroscedasticity (*Lagrange Multiplier* = 12.311, *degrees of freedom* = 4, *p* = 0.015). Multicollinearity was not a concern, with VIFs ranging from 1.052 to 1.496. The largest Cook’s distance was 0.074, indicating that no individual observation exerted undue influence on the regression estimates. Although 21 observations had leverage values greater than 4/n, excluding these observations did not materially change the direction or statistical interpretation of the main associations. Sensitivity analyses using HC3 heteroscedasticity-consistent standard errors yielded the same substantive conclusions as the primary analyses.

## 4. Discussion

### 4.1. Descriptive Characteristics of SV Experience, Psychological Resilience, and Professional Identity in Nurses Following NAEs

Compared with Li et al.’s findings among ICU nurses, the SV experiences reported by nurses in this study appeared less severe [23]. This finding may reflect differences in event types and acuity across settings; the present sample was dominated by patient falls and catheter dislodgement, whereas ICU samples may include more acute events such as medication errors or cardiac arrests. Even seemingly common NAEs may have lasting psychological consequences for the nurses involved. It is worth noting that, in this study, psychological distress yielded the highest mean item score among the SVEST distress dimensions, a finding consistent with Nan et al. [24]. This pattern may be associated with the predominance of non-life-threatening NAEs (e.g., patient falls and catheter dislodgement) and comparatively limited reported impairment in physical and practitioner functioning. Nevertheless, as SVs, these nurses reported substantial distress arising from perceived inadequate support—particularly those practicing in tertiary Grade-A hospitals—suggesting that support deficits, rather than event severity per se, may be more closely associated with psychological distress in this population [25]. Therefore, in addition to providing psychological interventions targeting SVs’ distress following NAEs, support should be delivered across three levels: colleague support, management support, and family or friend support, with the aim of addressing the multifaceted needs associated with such events. Nurses who experienced SV in this study demonstrated a comparatively medium level of professional identity, consistent with prior findings yet exceeding the levels reported by Zhang et al. among psychiatric nurses [26,27]. This discrepancy may be attributable to the distinctive mental health challenges inherent in psychiatric settings, which tend to erode nurses’ professional identity. Furthermore, the excessive workload and associated occupational pressure in psychiatric nursing may exacerbate this effect. Consequently, targeted attention to the professional identity of nurses across different clinical departments is warranted. Nurses in our sample exhibited a marginally lower level of psychological resilience compared with that reported by Wei et al. among surgical nurses [28]. Several factors may account for this discrepancy. First, the participants in our study were younger on average, and younger age has been consistently associated with lower resilience due to less accumulated coping experience and emotional regulation skills. Second, our sample was recruited from provincial Grade-A Tertiary hospitals, where nurses face prolonged high workloads. Such chronic occupational stressors may also be associated with lower emotional resources and resilience. Collectively, these contextual differences support further evaluation of interventions designed to strengthen resilience among younger nurses in high-intensity settings.

### 4.2. Association Between SV Experience and Psychological Resilience as Well as Professional Identity

In the present study, both psychological resilience and professional identity were negatively associated with nurse’s SV experiences of NAEs, while psychological resilience was positively associated with professional identity. These findings indicate that the three variables covaried in this cross-sectional sample, without establishing their temporal order. Our results align with Guo et al. [29], who reported that greater post-event support correlated with less severe negative SV experiences and higher psychological resilience. Thus, institutional support may be a relevant correlate, although a causal buffering effect cannot be inferred. More severe SV experience was also associated with lower professional identity, consistent with Luo et al. [30]. These cross-sectional findings are compatible with the possibility that the psychological impact of NAEs is linked to fundamental professional self-conception in addition to immediate distress. The positive association between psychological resilience and professional identity corroborates the findings of Xia et al. [31]. One possible interpretation is that higher psychological resilience is observed alongside greater adaptive capacity when nurse experience NAEs, with concurrent enhancement of professional skills and service quality coinciding with stronger professional identity in the context of demonstrated competence and continued growth; these cross-sectional patterns are compatible with the view that resilience and adaptive professional functioning cooccur after NAEs, but reverse or reciprocal associations remain possible. When the two SV experience dimensions were examined separately, both SV distress and perceived support deficit were associated with lower psychological resilience. This finding suggests that post-event adaptation is influenced by both what nurses experience internally and what they perceive in their surrounding environment. Perceived support deficit showed the most stable association with professional identity. This pattern suggests that the response received after an NAE may influence how nurses interpret not only the event, but also their place within the professional community. The association between support deficit and professional identity remained after distress was considered. This is noteworthy because it suggests that the support environment may have relevance beyond the immediate relief of emotional discomfort. Perceived support may be linked to professional recognition, fairness, and belonging—elements that are central to professional identity [32]. Psychological resilience should therefore be considered a potentially important correlate and a candidate for longitudinal and intervention research, rather than a protective mechanism established by the present study.

### 4.3. Psychological Resilience Mediates the Relationship Between SV Experience and Professional Identity

The mediation analysis showed that the statistical role of psychological resilience differed across the total SV experience score and its dimensions. For the total SV experience score, the indirect effect through psychological resilience was significant whereas the direct effect was not. This pattern is consistent with the view that nurses’ professional identity is related not only to the severity of NAEs but also to their capacity for psychological adaptation when facing such experiences [33]. The total SV experience score should be interpreted with caution, as it combines distress-related dimensions with reverse-coded support dimensions. In our sample, distress and support deficit were essentially uncorrelated, suggesting that the total score may not reflect a single unified construct but rather multiple distinct aspects of the SV experience. Therefore, the dimension-level findings may provide a more meaningful interpretation. For the support dimension, both the indirect effect through psychological resilience and the direct effect were significant. The support dimension appears to involve both individual and contextual features. On the one hand, perceived support is closely related to psychological resources and may therefore be reflected in psychological resilience. On the other hand, support also carries interpersonal and organizational meaning, including collegial recognition, team climate, and the degree of institutional responsiveness after NAEs [34]. This may help explain why a significant direct association remained even after psychological resilience was included in the model. For the distress dimension, the indirect effect was significantly negative, the direct effect was significantly positive, and the total effect was not significant. This pattern suggests that distress may relate to professional identity through two opposing pathways. A plausible interpretation is that distress may indirectly diminish professional identity by eroding psychological resilience, while simultaneously being directly associated—under certain conditions—with greater professional engagement or heightened responsibility awareness. Similar suppression effects have been observed in occupational stress research, where distress can both deplete personal resources and motivate compensatory efforts [35]. However, given the cross-sectional design, the selected nature of the sample (requiring self-reported distress for eligibility), and the multidimensional structure of the SVEST, this suppression pattern could also arise from statistical conditioning or conceptual overlap between constructs. Therefore, we emphasise that the proposed explanation should be tested in future longitudinal or experimental studies. The coexistence of these opposing associations may account for the suppression pattern and the non-significant total effect. Overall, these results suggest that different aspects of SV experience may correspond to different psychological resilience and professional identity correlates and therefore should not be interpreted as a uniform construct. Given the cross-sectional design, the observed mediation should be understood as statistical mediation rather than directional or causal mediation.

These findings carry significant implications for nursing management and clinical practice. This study extends the literature by examining psychological resilience in relation to the associations between SV experience and professional identity and, importantly, by showing that this statistical pattern differed across the total score and specific dimensions of SV experience. For the overall SV experience score, psychological resilience accounted for most of the observed association with professional identity, whereas for the support and distress dimensions, the pattern was more complex. This suggests that psychological resilience is an important factor in understanding how SV experience relates to nurses’ professional identity, but it also underscores that SV experience encompasses more than just distress. It involves multiple dimensions, including perceived support, which may interact differently with resilience. Therefore, it is crucial to distinguish between these distinct aspects of SV experience in both practice and research, rather than treating the overall score as a single, homogeneous exposure. From a practice perspective, these findings tentatively point to several factors that may warrant attention in nursing management and organisational support. First, perceived support deficit—reflecting collegial, managerial, and social support—showed association with lower professional identity, suggesting that support-related factors deserve consideration in efforts to understand nurses’ professional outcomes following NAEs. Second, psychological resilience appears to be a relevant factor in this association; however, the dimension-specific results indicate that resilience alone may not capture the full picture, and that interpersonal and organisational conditions—such as team climate, managerial responsiveness, and institutional recognition following NAEs—may also warrant separate attention. Third, the suppression pattern observed for the distress dimension suggests that distress-related responses should be interpreted cautiously and not assumed to have a uniform professional meaning, pointing to the importance of considering both the potential negative and positive aspects of distress responses when examining how nurses adapt to NAEs. Similarly, approaches such as peer support, mentorship, reflective debriefing, and team-based interventions are potential avenues for exploration, but their relevance to professional identity outcomes remains to be established in future research [36,37]. The present findings suggest that psychological resilience warrants continued attention in research and nursing support practice, while also underscoring the importance of addressing the broader support context in which SV experiences are embedded. Overall, because these findings are derived from a cross-sectional study with a selected sample, they should be viewed as hypothesis-generating rather than practice-changing. Longitudinal and interventional studies are needed to examine whether the observed patterns of association remain stable over time and whether the factors identified in this study are indeed modifiable.

## 5. Conclusions

Among nurses experiencing NAEs, SV experience, psychological resilience, and professional identity were statistically associated. However, the statistical role of psychological resilience differed across SV dimensions. For the total score, the indirect effect through psychological resilience was significant, while the direct effect was not. In contrast, for the support dimension, both the direct and indirect effects were statistically significant. In the distress dimension, the indirect effect through psychological resilience was significant but showed an opposing direction relative to the direct effect, resulting in a non-significant total effect. These findings indicate that SV experience should not be treated as a uniform construct when examining its relationship with professional identity. Rather, distinct dimensions appear to correspond to different statistical correlates and processes. Psychological resilience remains a relevant factor in this context, but the present cross-sectional patterns should be understood as statistical associations rather than evidence of causal mediation.

Several limitations should be acknowledged. First, the cross-sectional design precludes determination of causal relationships or temporal ordering. We cannot rule out reverse causation, bidirectional relationships, or unmeasured confounding. Longitudinal or prospective cohort studies are needed to clarify the temporal sequencing among these variables. Second, data were collected from a single tertiary hospital using convenience sampling, and only nurses directly experienced an NAE who self-reported related distress were included. This selection criterion may limit the generalisability of our findings and may have reduced variability in distress scores, given that distress was both an eligibility requirement and a component of the exposure measure. Such range restriction may have attenuated the associations for the distress dimension and may have contributed to the opposite signs of the indirect and direct effects observed in the distress model. Future multi-centre studies with broader sampling strategies that include all nurses experienced NAEs, regardless of self-reported distress, are needed to better characterise the SV experience. Third, the use of self-report measures may have introduced recall bias, social desirability bias, and common-method variance. Future studies should consider incorporating objective measures or mixed-methods approaches. Furthermore, the lack of a comparison group without SV experience limits the ability to assess the unique contribution of SV experience to the observed associations. The dimension-specific mediation patterns observed here may also be context-specific; replication across diverse healthcare environments and cultural backgrounds is warranted. Future research should further explore how event-related characteristics—such as event severity, temporal proximity, organizational response quality, and cumulative exposure history—intersect with SV dimensions, psychological resilience, and professional identity.

This study examined the statistical associations among SV experience, psychological resilience, and professional identity in nurses experiencing NAEs, revealing that the role of resilience varies across the total score and specific dimensions of SV experience. These findings enrich current understanding of professional identity in the context of SV experiences and highlight the value of dimension-specific inquiry. From a practical perspective, the results suggest that both resilience-related support and broader organizational support structures may be meaningful in nurse support programs, education, and counseling services, particularly for nurses with greater SV experience and those in potentially vulnerable subgroups. Overall, this study provides preliminary, cross-sectional evidence that may inform future longitudinal, multi-center, and methodologically diverse research.

## Figures and Tables

**Table 1:** The Demographic Characteristics and Professional Identity Scores of SV Nurses (*n* = 368).

General Data	Groups	N (%)	Professional Identity (M ± SD)	*F/t*	*p*
Gender	Male	16 (4.35)	117.12 ± 20.43	0.447	0.655
	Female	352 (95.65)	114.64 ± 21.82		
Age (Years)	≤28	79 (21.47)	113.23 ± 18.97	1.565	0.197
	29–33	164 (44.57)	114.90 ± 22.66		
	34–38	98 (26.63)	113.44 ± 21.45		
	≥39	27 (7.34)	123.04 ± 23.88		
Marital status	Single	115 (31.25)	113.04 ± 19.50	0.786	0.456
	Married	248 (67.39)	115.67 ± 22.70		
	Others	5 (1.36)	108.40 ± 22.83		
Religion belief	Yes	27 (7.34)	114.22 ± 24.56	−0.130	0.897
	No	341 (92.66)	114.79 ± 21.54		
Academic degree	Associate	20 (5.43)	120.80 ± 26.65	0.826	0.439
	Bachelor	338 (91.85)	114.42 ± 21.43		
	Master or above	10 (2.72)	113.60 ± 22.19		
Professional title	Junior	176 (47.83)	114.01 ± 20.94	1.956	0.143
	Intermediate	183 (49.73)	114.77 ± 22.43		
	Senior	9 (2.45)	128.67 ± 20.30		
Employment status	Employed by department	213 (57.88)	113.97 ± 21.08	2.233	0.109
	Employed by hospital	152 (41.30)	115.34 ± 22.56		
	Authorized	3 (0.82)	140.00 ± 12.29		
Working period (Years)	≤8	185 (50.27)	113.58 ± 20.67	2.052	0.130
	9–15	143 (38.86)	114.45 ± 23.02		
	≥16	40 (10.87)	121.20 ± 21.30		
Type of NAEs	Fall	162 (44.02)	114.55 ± 21.99	0.820	0.555
	Catheter slipping	128 (34.78)	114.52 ± 21.73		
	Medicine misuse	9 (2.45)	103.22 ± 26.44		
	Self-harm	12 (3.26)	120.67 ± 22.40		
	Burning or scalded	5 (1.36)	107.60 ± 22.21		
	Medicine leakage	22 (5.98)	115.41 ± 19.97		
	Others	30 (8.15)	118.57 ± 20.25		
Time of NAEs occurred	Second half of 2023	121 (32.88)	115.07 ± 22.08	1.962	0.084
	First half of 2023	77 (20.92)	114.43 ± 21.59		
	Second half of 2022	61 (16.58)	108.43 ± 18.67		
	First half of 2022	55 (14.95)	115.78 ± 21.88		
	Second half of 2021	33 (8.97)	118.88 ± 24.11		
	First half of 2021	21 (5.71)	123.19 ± 21.92		
psychological counseling	Yes	65 (17.66)	123.08 ± 23.19	3.455	<0.001
	No	303 (82.34)	112.96 ± 21.03		

Note: SV, second victim; NAE, nursing adverse event; M ± SD, mean ± standard deviation.

**Table 2:** The scores of SVEST (*n* = 368).

Dimensions	Scores ($\bar{x}$ *± s*)	Average ($\bar{x}$ *± s*)
Psychological distress	14.83 ± 3.62	3.71 ± 0.90
Physical distress	7.46 ± 3.16	2.49 ± 1.05
Practitioner distress	12.04 ± 5.20	2.41 ± 1.04
Colleague support	5.65 ± 2.08	1.88 ± 0.69
Management support	10.77 ± 4.30	1.54 ± 0.61
Family or friend support	4.28 ± 2.09	2.14 ± 1.05
Total score of SVEST	55.03 ± 12.42	/

Note: SVEST, Second Victim Experience and Support Tool.

**Table 3:** The scores of PIS and CD-RISC (*n* = 368).

Dimensions	Scores ($\bar{x}$ ± *s*)
Professional cognitive evaluation	33.51 ± 7.02
Professional social support	23.47 ± 4.20
Professional social skills	21.51 ± 4.69
Professional frustration response	23.32 ± 4.46
Professional self-reflection	12.01 ± 2.20
Total score of PIS	113.82 ± 21.42
Total score of CD-RISC	28.04 ± 7.33

Note: PIS, Professional Identity Scale for Nurses; CD-RISC, Connor–Davidson Resilience Scale.

**Table 4:** The association between SV Experience and psychological resilience as well as professional identity (*n* = 368).

	CD-RISC	Cognitive Evaluation	Social Support	Social Skills	Frustration Response	Self-Reflection	PIS
Colleague support	−0.322 **	−0.347 **	−0.429 **	−0.303 **	−0.383 **	−0.397 **	−0.384 **
Management support	−0.444 **	−0.514 **	−0.501 **	−0.399 **	−0.556 **	−0.582 **	−0.529 **
Family/friend support	−0.243 **	−0.401 **	−0.421 **	−0.346 **	−0.386 **	−0.361 **	−0.407 **
Practitioner distress	−0.402 **	−0.209 **	−0.196 **	−0.187 **	−0.270 **	−0.319 **	−0.236 **
Psychological distress	−0.149 *	0.057	0.094	−0.003	0.061	0.044	0.054
Physical distress	−0.291 **	−0.032	−0.034	−0.052	−0.083	−0.118	−0.058
SVEST	−0.534 **	−0.383 **	−0.379 **	−0.339 **	−0.438 **	−0.479 **	−0.414 **
CD-RISC	1	0.612 **	0.600 **	0.637 **	0.612 **	0.674 **	0.661 **

Note: * *p* < 0.05, ** *p* < 0.01. SV, Second Victim; SVEST, Second Victim Experience and Support Tool; PIS, Professional Identity Scale for Nurses; CD-RISC, Connor–Davidson Resilience Scale.

## Data Availability

The datasets generated and/or analyzed during the current study are not publicly available due to ethical restrictions and participant privacy protections. De-identified data may be available from the corresponding author upon reasonable request, subject to ethics approval and institutional permissions.

## Abbreviations

The following abbreviations are used in this manuscript:

B	Unstandardized Coefficient
CI	Confidence Intervals
M ± SD	Mean ± Standard deviation
NAE	Nursing Adverse Event
PIS	Professional Identity Scale for Nurses
STROBE	Strengthening the Reporting of Observational Studies in Epidemiology
SV	Second Victim
SVEST	Second Victim Experience and Support Tool
SPSS	Statistical Product Service Solutions
SE	Standard Error
VIF	Variance Inflation Factor
β	Standardized Regression Coefficient
